# On the Organization of the Medical Profession

**Published:** 1847-08

**Authors:** 


### PART V.—EDITORIAL.

ARTICLE I.

### ON THE ORGANIZATION OF THE MEDICAL PROFESSION.

While physicians complain of the want of discrimination exhibited by the public in relation to the qualifications of medical men, whereby the ignorant are enabled to gain an extensive practice, and the better qualified are compelled to languish in neglect, they seem, for the most part, to forget that in cases where their own influence is concerned they have done nothing to remove so great an evil, but rather have aided in its perpetration. We allude now especially to the election of medical professors and the bestowing of patronage upon the schools to which they are attached without requiring any certain or public proof of fitness for such stations. These, and the situations of surgeons and physicians to hospitals are, under our present arrangments, the highest positions in the profession to which we can aspire, and nothing seems more reasonable than that they should be offered as rewards for the successful cultivation of science, and be open to any aspirant to professional eminence. But instead of this what do we see? The appointing power to these places is, in the United States, vested in about thirty-seven corporate bodies composed mostly of non-medical men ignorant of what constitutes a good professor, and of the relative standing of the various candidates who may present themselve. The greater number of these bodies are directly connected with some religious denomination, and think themselves bound te act for its advancement. What would be thought of an ecclesiastial body dependent for their election upon a body of lawyers or doctors? How could teachers of law, judges of courts, or high officers of any kind be chosen by corporate bodies, yet neither of these things would be more absurb than the present method of choosing medical professors. Those who are to choose have no certain means of judging of qualification. Those who are to be chosen have no means of demonstrating their ability to teach. Candidates find that to cultivate the acquaintance of influential individuals is a more successful method of succeeding than to spend time in prosecuting scientific researches. Sectarian influences frequently prevail, and those of a personal nature are never entirely excluded. The hopelessness of success prevents most persons from making application, and acts as a practical exclusion of the great mass of the profession. The method recently proposed of advertising for applications and keeping them secret, while it proves that necessity for change has been felt, is insufficient, as it does not enable a correct judgment of the qualifications of a candidate to be formed.

Whence comes this absurd system by which those most interested and most capable of judging, are the only ones excluded from a voice in the choice of professors? It is a remnant of a system of government inherited from another country and from a half barbarous age. While in later times the vivifying effect of liberal institutions has been shed upon the mass of the population exciting them to activity and ambition, while exclusive privileges have been abrogated as pernicious, while the various professors and teachers have been called upon to make rules for their own government, the medical profession in our own country has slumbered under the forms of a past century. With no common head, without means of communication among its different parts, without association or organization, what could be expected but that it should exhibit, as it does at the present moment, a perfect image of chaos.

To centralize, to harmonize, to bring the different parts into communication with each other, to stimulate to activity the great body of practitioners and interest them in the progress of science—such are the first and most urgent wants of the medical public. They have been generally felt, and the assembling of a national convention, and the organization of a national association is the first step towards their relief. The action of this association thus far has been judicious and its influence salutary. But it will be of little avail unless the interests of the entire profession shall be represented and its wishes expressed. It is for this reason that we earnestly call upon the physicians of the north west to organize themselves into associations for improvement and to have their representatives at the next meeting in Baltimore, instructed to vote for all measures calculated to elevate the profession but especially to call upon medical schools to adopt some method by which the merits of candidates for teachers' chairs shall be fairly tested by public trials. It is especially to physicians of this region that we look for such action ; as they are untrammelled by vicious forms and free to act for the public good without coming in conflict with long established interests.

It is not necessary for us to enumerate, at present, all the different beneficial effects likely to result from the adoption of this system, or to answer all the objections which might be urged against it. It has a merit which its opponents can not deny, and what is in itself sufficient to secure its triumph, it is a perfect security against the success of persons who are incompetent or who have been guilty of disgraceful conduct. If any still have doubts upon the subject let them look at the system of *concours* and its effects in France and they will be speedily removed.

Lest some might suppose, from our frequent recurrence to this subject, that we are unduly attached to this "one idea," we will say in addition, that we regard it as but one of several improvements necessary to the proper improvement of the profession. It is scarcely necessary to add that we have no personal interest or feeling on the subject except what springs from a wish to subserve the interests of mankind at large. As at present constituted, the medical organization might justly be compared to that of inferior animals, where separate ganglia, remotely connected by nervous cords, support a moderate degree of vitality independent of each other for which state of imperfect life, we desire to see substituted one in which there shall be a nervous centre, active communication and mutual dependence between the different parts; in a word, that perfect state of development in which modern society reflects the image of man himself. D. B.

